# A new era of allele-specific diagnostics?

**DOI:** 10.3389/fgene.2013.00134

**Published:** 2013-07-18

**Authors:** Arthur J. Lustig, Antonella Sgura

**Affiliations:** ^1^Department of Biochemistry and Molecular Biology, Tulane University Medical CenterNew Orleans, LA, USA; ^2^Department of Science, University of “Roma Tre”Rome, Italy

From the accompanying paper (Djansugurova et al., 2013), it is clear that cancer screening is making a global impact in reducing cancer mortality, a major advance that will increase in value as more therapeutic regimens become available on a worldwide basis. The genes of value are ones in which specific alleles are statistically related to oncogenesis. The major arbitrary element is a potential bias in the functionality of the specific panel of alleles of the gene chosen to use for regional screening. In almost all cases, the choices are drawn upon from the literature by their frequency of oncogenic phenotypes, rather from the potential mechanism of the defect (Ginsberg et al., 2011).

There has been a disharmonious relationship between diagnostics and molecular biology, with most molecular biologists feeling that these diagnostic studies do not lead to mechanistic information. However, based on the studies such as those described here (Djansugurova et al., 2013) (e.g., Schneider et al., 2008), there is an increasing potential for diagnostic studies to provide information on the nature of steps toward oncogenesis and to put these studies and others into a molecular context. For this to occur, a familiarity with the gene product, and the allelic phenotype will lead to the most specific screens and paths to oncogenesis.

In this study, three groups of genes were chosen based on basic possible correlative elements gleaned from the literature. Although defects are present in the “sledgehammer” genes that are involved in the detoxification of cells, mutations in these would be bound to have pleiotropic and deleterious effects without really telling us much about the mechanism.

Hence, our interest lies in the two remaining groups, part of the XRCC class of genes influencing DNA damage repair. The author states erroneously that the XRCC1 gene is involved in DNA DSB response, although the careful analyses have revealed otherwise (Martinez-Macias et al., 2013). The confusion as to pathway is likely to be due to the possible processing of base pair gaps into double stranded breaks, albeit that is not their primary defect. Indeed, XRCC1 is a scaffold protein that enhances the C methylation of bases and interacts in a domain-specific fashion with the repair associated proteins, including APE, PARP, and Pol β, preventing the Ogg1-mediated single stranded nick formation via an efficient break excision repair (BER). BER effectively prevents abasic site single stranded gap formation (Mourgues et al., 2007). And, hence, genome stability. Consistent with these data, the homologous yeast Ogg1 protein methylates telomeric DNA under oxidative stress through the creation single stranded substrates for the BER machinery (Lu and Liu, 2010). Clearly, within Kazakhstan, the abasic damage of DNA is sufficient to initiate the process that ultimately leads to oncogenesis. The prediction would be that the downstream oncogenes contain a vast number of mutations caused by these defects.

The study of XRCC1 with the identified specific alleles will undoubtedly start to allow the analysis of a regulator that ultimately influences BER. The phenotype of alleles at the molecular level will be able to define the domain structure of the protein and the pathway leading to mechanism of repairing abasic DNA gaps.

However, most studies use the same panel of mutations, which may be diagnostically fruitful, but be mechanistically limiting (Ginsberg et al., 2011). The diagnostic value of more complex protein variants that may bind to more factors may be problematic.

Importantly, the genes that have the predominant effect may not be disease-specific. Rather, they may represent a population-based clustering of genes and alleles in specific populations and may vary among differing populations. Nonetheless, this study does contain some intriguing mechanistic hints that can be gleaned from the specific alleles that are defective in esophagitis and cervical cancer.

Indeed, an allele of XRCC3, a paralog of the Rad51 DSB gene, that coats the invading DNA strand did not have an influence on esophageal and cervical cancer in this region. These data suggest the absence of double strand breaks (DSB) or checkpoints involved in DSB repair that can create a break that can be resolved by end ligation, homologous recombination with ectopic repeats, and a potential site for transposition (Liu et al., 2007). Normally, such a break would lead to the activation of the ATM or ATR checkpoint, and the cascade of repair enzymes. If this pathway fails, a checkpoint will be initiated in the cell cycle to stop growth. Overcoming this checkpoint is indeed a road to oncogenesis. These data suggest that, in this population, overall DSB-initiated repair and checkpoint control are not involved in the etiology of the disease. Similarly, the absence of differences in mutations in TP53 (Wahl et al., 1997), heavily involved in double strand break repair and telomere checkpoint control, show no significant differences among healthy and patient populations. Similarly, a more removed kinase, cyclin D1 that regulates the timing of entry into the cell cycle does not influence the frequency of disease in this region.

Hence, we are left with a testable hypothesis: that inducers of mutagenesis by BER defects are involved in the etiology of the two cancers studied in this region. These genes are likely to give rise to oncogenic states as mutagens with a multiplicity of targets capable of producing nonsense codons and missense mutations. The specific targets are likely to be genes involved in the regulation of growth control, as a secondary effect of the inducers. There are likely to be a great deal more information that will be derived from mutations within other DNA repair-associated genes, some of which could be hypomorphic or haplo-insufficient alleles, in the near future.
